# Characterization of Human CD8αβ Interaction With Classical and Unconventional MHC Molecules

**DOI:** 10.1002/eji.202451230

**Published:** 2024-12-20

**Authors:** Ben de Wet, Robert Alan Simmons, Richard J. Suckling, Rita Szoke‐Kovacs, Salah Mansour, Marco Lepore, David K. Cole, Jakub Jaworski, Alexandra L. Chapman, Milos Aleksic

**Affiliations:** ^1^ Immunocore Limited Abingdon Oxon UK; ^2^ NIHR Biomedical Research Centre, School of Clinical and Experimental Sciences, Faculty of Medicine University of Southampton Southampton UK; ^3^ Institute for Life Sciences University of Southampton Southampton UK; ^4^ Versant Ventures/Ridgeline Discovery Basel Switzerland

**Keywords:** TCR, CD8‐co‐receptor, T cell antigen recognition, K_D_, Surface Plasmon Resonance, thymic selection

## Abstract

The CD8 co‐receptor exists as both an αα homodimer, expressed on subsets of specialized lymphoid cells, and as an αβ heterodimer, which is the canonical co‐receptor on cytotoxic T‐cells, tuning TCR thymic selection and antigen‐reactivity in the periphery. However, the biophysical parameters governing human CD8αβ interactions with classical MHC class I (MHCI) and unconventional MHC‐like molecules have not been determined. Using hetero‐dimerized Fc‐fusions to generate soluble human CD8αβ, we demonstrate similar weak binding affinity to multiple different MHCI alleles compared with CD8αα. We observed that both forms of CD8 bound to certain alleles with stronger affinity than others and found that the affinity of thymically selected TCRs was inversely associated with the affinity of the CD8 co‐receptor for the different alleles. We further demonstrated the binding of CD8αα and CD8αβ to the unconventional MHC‐like molecule, MHCI‐related protein 1, with a similar affinity as for classical MHCI, but no interaction was observed for the other unconventional MHC‐like molecules, CD1a, b, c, or d. In summary, this is the first characterization of human CD8αβ binding to MHCI and MHC‐like molecules that revealed an intriguing relationship between CD8 binding affinity for different MHCI alleles and the selection of TCRs in the thymus.

## Graphical abstract

Human CD8αβ was produced as an Fc‐fusion via knob‐in‐hole mediated heterodimerization and bound class I pHLA and MR1 with similar affinity to human CD8αα, but did not bind CD1a, b, c, or d. An inverse relationship between CD8:pHLA affinity and TCR:pHLA was observed for several alleles.

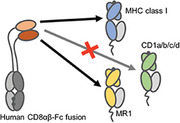

## 1 Introduction

Cytotoxic T‐cells play a key role in the immune response by recognizing and killing virally infected and transformed cells. Antigen recognition is mediated by binding of the T‐cell receptor (TCR) to antigenic peptides displayed on class I MHC (pMHCI) on the surface of target cells with the concomitant engagement of the co‐receptor CD8. MHCI:CD8 binding stabilizes the resultant tripartite TCR:pMHCI:CD8 complex [1] and brings the CD8‐associated Src‐family kinase, Lck, in close proximity to phosphorylatable immunoreceptor tyrosine activation motifs (ITAM) found in the cytoplasmic tails of TCR‐CD3 complex, a crucial first step in T‐cell triggering [2]. CD8 is found as an αβ‐heterodimer on cytotoxic T‐cells and as an αα‐homodimer on γδ T‐cells, intraepithelial lymphocytes, NK cells, and subsets of dendritic cells [3]. Each CD8 monomer consists of an Ig‐like domain followed by an extended unstructured stalk region and a transmembrane domain. The Ig‐like domains contain CDR‐like loops that, in the case of murine CD8αβ, engage with the α3‐domain of MHCI, while human and murine CD8αα CDR‐like loops additionally contact the α2‐domain and β2m. It has been proposed that this mode of engagement along with the conserved binding orientation of TCR:pMHC interaction ensures the close localization of Lck with the CD3 complex during TCR triggering [4].

Murine CD8αα and CD8αβ have been expressed in soluble form while only soluble CD8αα from humans has been successfully produced. Reported solution affinities of CD8 for MHCI alleles are weak and differ for different MHCI alleles, with most reported K_D_’s between 100 and 220 µM for the human MHCI:CD8αα interaction, and even K_D_’s in the millimolar range have been measured for certain alleles such as HLA‐A*6801 [5]. By comparison, the reported affinities of murine CD8αα and CD8αβ for pMHCI are stronger with K_D_’s between 7 and 200 µM, and 14 and 135 µM, respectively [4]. The weak affinity of CD8 for pMHCI likely has functional relevance, as increasing the affinity of the interaction leads to TCR‐independent triggering [6]. Furthermore, the contribution of CD8 to T cell activation is dependent on the strength of the TCR:pMHCI interaction, with weaker agonists unable to trigger T cells in the absence of CD8 binding and with T cell activation only mildly impaired by a lack of CD8 binding in the case of stronger agonists [7]. Affinity of TCR for self‐pMHC is modulated by negative and positive selection in the thymus in the presence of CD8 co‐receptor and results in a pool of circulating T‐lymphocytes with weak affinity for self‐pMHC [8] and it has been shown that CD8 co‐receptor scanning imposes dwell‐time and consequently affinity constraints on the self TCR‐pMHCI interaction [9].

In addition to conventional CD8^+^ cytotoxic T‐cells that bind pMHCI, there are innate‐ and adaptive‐like subpopulations of CD8+ lymphocytes that respond to non‐peptide antigens displayed on MHC‐like molecules with a very similar overall structure to conventional MHCI [10]. Lipid antigens are presented by the four isoforms of CD1 (a, b, c, and d), each with different ligand specificity [3]. MHCI‐related protein 1 (MR1) presents bacterially‐derived riboflavin metabolites and its structure in complex with CD8αα as well as the role of this interaction in binding and activation has recently been reported [11].

Here, we demonstrate the first successful expression of soluble human CD8αβ and analysis of its binding to several classical pMHCI alleles as well as MR1 and CD1, in comparison to soluble human CD8αα. We further demonstrate an inverse relationship between the affinity of CD8 and TCR for pMHCI alleles, a characteristic that is likely a functional consequence of thymic selection.

## 2 Results and Discussion

### 2.1 Expression of Soluble Human CD8

To date, successful attempts to express soluble human CD8αβ have not been reported and its binding characteristics remain unexplored. In our study, the production of human CD8αβ was achieved by fusing either of the CD8αβ polypeptides to distinct IgG1 Fc‐regions that contain complementary mutations promoting heterodimerization [12]. CD8αα was similarly produced, fused to the IgG1 Fc‐region, but lacking hetero‐dimerization motifs. This allowed for the expression of the complete extracellular domains of CD8αβ and CD8αα in mammalian cells and purification using affinity methods (Figure S1).

### 2.2 Molecular Investigation of CD8αβ and CD8αα Interactions With Classical and Unconventional Antigen Presenting Molecules

**FIGURE 1:**
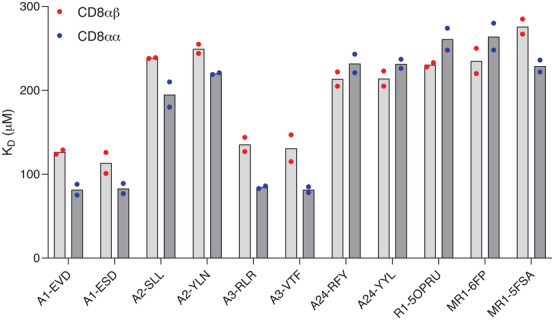
CD8 shows differential binding to different MHCI alleles and MR1. Equilibrium dissociation constants of CD8αβ‐Fc (light grey bars with red circles) and CD8αα‐Fc (dark grey bars and blue circles) binding to a number of MHCI alleles with different peptides and MR1 loaded with different cargoes, measured by SPR. Measurements were performed at 25°C and KD‐values are duplicate experimental repeats.

The binding of CD8αβ and CD8αα to antigen‐presenting molecules carrying different ligands was studied by SPR (Figure 1, Table S1, Figure S2–S4). Affinities of CD8αβ and CD8αα for MHCI were weak, with equilibrium dissociation constants (K_D_’s) in a similar range (80–250 µM) as previously reported [4]. Both isoforms of CD8 showed distinct affinities for different MHCI alleles, with CD8αα generally binding with a slightly stronger affinity to a particular allele compared with CD8αβ. Affinities of CD8αβ and CD8αα for HLA‐A*01:01 and HLA‐A*03:01 were higher (K_D_ 80–120 µM) compared with HLA‐A*02:01 and HLA‐A*24:02 (K_D_ ∼230 µM) (Table S1) (hereafter HLA‐A1, HLA‐A3, HLA‐A2, HLA‐A24). Very similar results were obtained when CD8αβ and CD8αα were immobilized on the chip surface and pMHCI was flowed as the analyte (Figure S2B, Table S2), indicating that the observed difference in affinities was not the result of spatial constraints in the system. The specificity of the interaction was further confirmed by the lack of binding of CD8 to HLA‐A2(D227K/T228A) that harbors mutations that prevent specific CD8 contacts [13] (Figure S2C). These binding data show that the weak affinity of human CD8αβ for MHCI, reported here for the first time, is a generally conserved feature of the T‐cell antigen recognition machinery, in agreement with observations for mouse CD8αα and CD8αβ and human CD8αα. The importance of this weak affinity has been highlighted by functional studies in which increasing the strength of the interaction leads to loss of specificity [14], and even antigen‐independent triggering [6], whereas abrogating the interaction leads to reduced sensitivity of the response [7].

**FIGURE 2:**
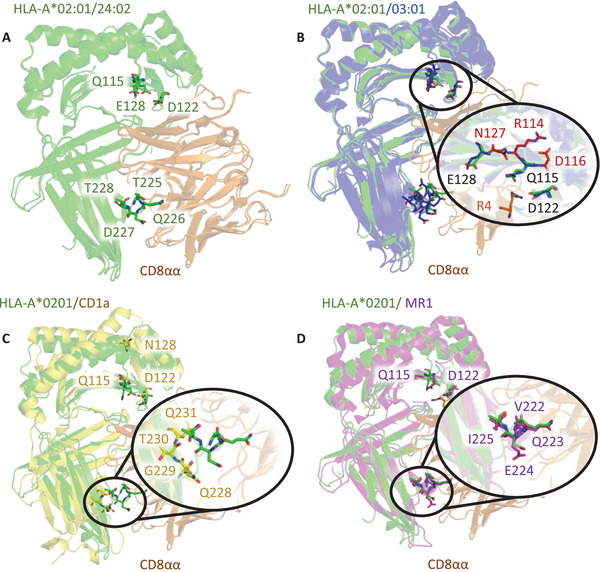
Structural modeling of CD8 binding to different antigen‐presenting molecules. A) Superposition of the HLA‐A2‐CD8 co‐complex structure (PDB accession code: 1AKJ) and HLA‐A24‐CD8 co‐complex structure (PDB accession code: 3QZW). CD8 is displayed as an orange cartoon and HLA is displayed as a green cartoon. Main HLA contacted residues are displayed as green sticks. B) Superposition of the HLA‐A2‐CD8 co‐complex structure (PDB accession code: 1AKJ) colored as in A) and HLA‐A3 (PDB accession code: 3RL1), displayed as a blue cartoon. HLA residues that contact CD8 are shown as sticks, with a zoom‐in of the HLAα2 domain CD8 contact zone shown in the black oval. Differences in the HLA‐A3 sequence compared with HLA‐A2 that might influence CD8 binding are shown as red sticks. C) Superposition of the HLA‐A2‐CD8 co‐complex structure (PDB accession code: 1AKJ) colored as in A) and CD1a (PDB accession code: 5J1A), displayed as a yellow cartoon, with residues that represent CD8 contact residues shown as sticks. A zoom‐in of the HLAα3 domain CD8 contact zone is shown in the black oval with a comparison of the HLA‐A2 residues that form the CD8 binding loop (green sticks) and equivalent CD1a residues (yellow sticks). D) Superposition of the HLA‐A2‐CD8 co‐complex structure (PDB accession code: 1AKJ) colored as in A) and the MR1‐CD8 co‐complex structure (PDB accession code: 7UMG), displayed as a purple cartoon, with residues that represent CD8 contact residues shown as sticks. A zoom‐in of the HLAα3 domain CD8 contact zone is shown in the black oval with a comparison of the HLA‐A2 residues that form the CD8 binding loop (green sticks) and equivalent MR1 residues labeled (purple sticks).

To further investigate the hierarchical binding of CD8 to MHCI alleles, we studied the sequences and structures of regions that are known to interact with CD8 (Figure 2A,B). The crystal structure of CD8αα with HLA‐A2 [15] shows that CD8 interacts primarily with the MHCIα3 domain, with some additional interactions with both the MHCIα2 domains and β2m. The primary CD8 contact residues in HLA‐A2 are Q115, D122, and E128 in the MHCIα2 domain and residues 225–228 (TQDT) in a flexible loop region in the MHCIα3 domain. Further important interactions are also made by residues in the MHCIα3 domain outside of this flexible loop at residues E198, E229, L230, V231, E232, and K243. Differences in the MHCIα3 domain are unlikely to be the main contributors to changes in the binding affinities for two reasons. First, although HLA‐A2 encodes a few differences in the MHCIα3 domain compared with HLA‐A24, HLA‐A1, and HLA‐A3, these differences are distal from the CD8 binding zone. Second, the MHCIα3 domains of HLA‐A24, HLA‐A1, and HLA‐A3 are identical, so the weaker affinity observed for HLA‐A24 compared with HLA‐A1/HLA‐A3 could not be explained by the difference in the MHCIα3 domain for these alleles. On inspection of the MHCIα2 domain, differences in residues between HLA‐A2/HLA‐A24 and HLA‐A1/HLA‐A3 were identified adjacent to residue Q115, which is known to influence CD8 binding [16] and E128. In HLA‐A2 and HLA‐A24, residue Q115 was flanked by H114 and Y116, and residue E128 was flanked by K127, whereas in HLA‐A1 and HLA‐A3, Q115 was flanked by R114 and D116, and E128 was flanked by N127. Thus, these differences may alter the flexibility or orientation of Q115, modulating the interaction with CD8. The role of residues flanking Q115 in modulating binding affinity was assessed by mutating these positions to the corresponding residues in either HLA‐A2 or HLA‐A1. Binding of CD8αβ to HLA‐A2(H114R/Y116D/K127) or HLA‐A1(R114H/D116Y/N127K) compared with wild‐type was assessed by SPR. Introduced mutations led to an increase in binding affinity to HLA‐A2 from a K_D_ of 189 to 146 µM and an analogous decrease in binding affinity to HLA‐A1 from a K_D_ of 107 to 257 µM (Figure S5), confirming structural predictions.

There are CD8^+^ lymphocyte populations able to respond to nonpeptide antigens such as lipids presented on CD1a, b, c, and d, and bacterial riboflavin metabolites presented on MR1 [3]. Recently, the structural basis for the CD8αα‐MR1 interaction was elucidated and CD8 was shown to contribute to MR1 reactivity [11], while CD8 binding to CD1 has not been reported. We tested the binding of CD8αβ and CD8αα to MR1 loaded with model ligands as well as CD1a, b, and c loaded with endogenous mammalian lipids and CD1d loaded with α‐galactosyl ceramide (α‐GalCer, KRN7000). Notably, neither CD8 isoform showed binding to any of the CD1 proteins despite measurable ILT2 binding [17], which verified the functional integrity of the immobilized CD1 ligands (Figure S4). Structural analysis demonstrated that, although some of the MHCIα2 domain residues that contact CD8 in classical MHCI were conserved in the CD1 molecules, their structural positions were not maintained, and most other residues that contact CD8 in the case of classical MHCI were different. Indeed, the flexible loop region in the MHCIα3 domain was substantially truncated in all the CD1 molecules compared with classical MHCI, ablating the main contact zone utilized by CD8 during binding (Figure 2C).

CD8αβ and CD8αα bound to MR1 with very similar affinities, with K_D_’s of the interaction around 250 µM (Figure 1, Figure S2, Table S1). The measured K_D_’s are comparable to the values obtained for CD8 binding to HLA‐A2 and HLA‐A24 and are in good agreement with the values reported for CD8αα bound to MR1, and the functional importance of CD8 binding in MR1 reactivity has recently been demonstrated [11]. The structural analysis demonstrated the conservation of the structural positions of residues Q115 and D122 in MR1 and MHCI, important in contacting CD8 for classical MHCI. Additionally, the overall structure and chemical nature of the flexible loop region in the MHCIα3 domain was very similar for both MR1 and classical MHCI. In particular, the roles of MHCI residues D227 and Q226 in forming the main contact interface between classical MHCI and CD8 were closely mimicked by MR1 residues E224 and Q223 (Figure 2D). Thus, the structural features of the MR1 α3 domain loop region, and interaction interface with CD8, were almost identical to the classical MHCI:CD8 interface providing a rationale for the similar binding affinities reported here, and previously [11], for both CD8αα and CD8αβ binding to MR1 and classical MHCI.

### 2.3 Inverse Relationship Between the Affinity of CD8 and TCR for pMHCI Alleles

**FIGURE 3:**
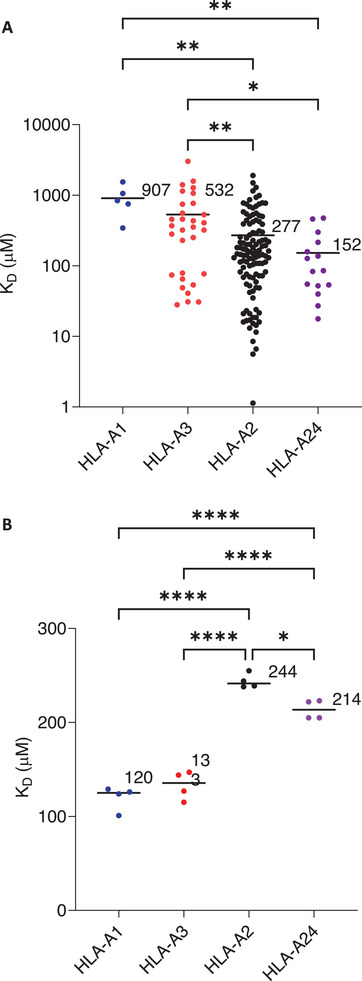
Inverse relationship between TCR:pMHCI and pMHCI:CD8 affinity. Equilibrium dissociation constants (KD) of (A) TCR:pMHCI binding measured for a number of thymically selected TCRs against peptides presented by HLA‐A1 (blue), HLA‐A3 (red), HLA‐A2 (black) and HLA‐A24 (purple) (single measurements of different pHLA's) compared with (B) pMHCI:CD8αβ binding measured for the same alleles (four experimental repeats for each allele). Averages are indicated next to the bar representing the same metric. One‐way ANOVA with correction for multiple comparisons was used with *p*‐value denoted by *p < 0.05, **p < 0.01, ***p < 0.001 and ****p < 0.0001.

Thymocytes expressing TCRs with weak affinity for self‐pMHC are positively selected in the thymus and CD4 or CD8 co‐receptor engagement is required during this process to ensure pMHC restriction [18]. Considering that CD8 binding contributes to the overall avidity of the tripartite complex during pMHCI engagement, it follows that the affinity of CD8 for different MHCI alleles may influence the affinity of the TCRs selected, depending on which allele/s they engage during thymic selection. We investigated this hypothesis by comparing the affinity of large number of in‐house generated, thymically‐selected TCRs for their cognate pMHCI targets (HLA‐A1–5 TCRs, HLA‐A2–116 TCRs, HLA‐A3–31 TCRs, HLA‐A24–15 TCRs), grouped by MHCI allele (Figure 3A) and compared the affinity of CD8 for the corresponding allele (Figure 3B). For the MHCI alleles tested, those with higher affinity for CD8 had on average a lower affinity for cognate pMHCI, and a variance‐weighted linear regression line fitted to pMHC:TCR K_D_ as a function of CD8αβ:TCR showed an inverse relationship with a coefficient of determination (*R* ^2^) of 0.71 (Figure S6). This observation is consistent with the suggestion that MHCI alleles with higher affinity for CD8 will result in the selection of TCRs with a correspondingly lower affinity for pMHCI of that particular allele.

### 2.4 Conclusion

Here, we report the first biophysical analysis of soluble human CD8αβ binding to several classical pMHCI alleles, as well as the unconventional MHC molecules MR1 and CD1, in comparison to soluble human CD8αα. These data demonstrate that (1) human CD8αβ and CD8αα bind with similar affinity to several different MHCI alleles, (2) human CD8 does not bind to CD1 molecules, (3) human CD8αβ binds to MR1 with a similar affinity to pMHCI, confirming its role in MAIT cell biology and MR1 reactivity, and (4) diversity in the MHCIα2 domain of classical MHCIs can potentially tune human CD8 affinity independently of the peptide cargo. Finally, we demonstrate an inverse relationship between the affinity of CD8 and TCR for pMHCI alleles, a feature that is potentially of functional importance during thymic selection.

## 3 Materials and Methods

### 3.1 Generation of CD8αβ‐Fc and CD8αα‐Fc Expression Plasmids

CD8 extracellular domains (CD8α—Uniprot P01732, residues 22–182 or CD8β—Uniprot P10966, residues 22–170) were cloned into pFUSE‐hIgG1e3 (Invivogen), downstream of a mouse kappa light‐chain secretion signal and upstream of an engineered human IgG1 Fc (IgG1 constant heavy chain [IGHG1] CH2‐ and CH3‐domains that had been modified with an IgG2 hinge region and substitutions to abrogate FcγRI‐binding). For expression of CD8αβ‐Fc heterodimers, IgG1 heavy chain CH3‐domains additionally carried complementary mutations (IGHG1‐Y290T in CD8α Fc‐fusion and IGHG1‐T249Y in CD8β Fc‐fusion) that favor heterodimerization [12]. Expression cassettes were subcloned into pcDNA3.1 and expressed under the control of the cytomegalovirus promoter.

### 3.2 Protein Expression and Purification

#### 3.2.1 Preparation of Soluble human CD8αβ‐Fc and CD8αα‐Fc

Soluble human CD8αβ‐Fc and CD8αα‐Fc fusions were produced using the ExpiCHO Expression System (ThermoFisher Scientific). ExpiCHO cultures were transformed with vectors encoding only CD8α‐Fc or CD8β‐Fc and CD8β‐Fc polypeptides and protein production allowed to proceed for 8 days. Supernatants were harvested, clarified and Fc‐fusions purified by applying to a 5 mL HiTrap protein A high‐performance column (Merck) and eluting with 0.1 M glycine pH 2.5 into 1 M Tris pH 9.5. Pooled, concentrated fractions were further purified by gel filtration into phosphate‐buffered saline (PBS) on Superdex 200 10/300 columns (Merck). The purified material was analyzed by SDS‐PAGE and Coomassie staining and quantified by measuring absorbance at 280 nm.

#### 3.2.2 Preparation of Biotinylated pMHCI

Human MHCI alleles, containing a C‐terminal Avi‐tag, and β‐2 microglobulin were expressed in *E. coli* and refolded from inclusion bodies with appropriate peptide/s (Peptide Protein Research) [19]. Resultant pMHCIs were purified by anion exchange and size exclusion chromatography and biotinylated using BirA ligase (Avidity).

#### 3.2.3 Preparation of Biotinylated MR1

Human MR1 containing a C‐terminal Avi‐tag, and β‐2 microglobulin were expressed in *E. coli* and refolded from inclusion bodies with various ligands [20]. 5‐(2‐oxopropylideneamino)‐6‐d‐ribitylaminouracil was produced by Gurdyal Besra (Birmingham University), 6‐formylpterin was from Schircks Laboratories, and 5‐formyl salicylic acid was from Merck. Ligand‐bearing MR1 was purified by anion exchange and size exclusion chromatography and biotinylated using BirA ligase (Avidity).

#### 3.2.4 Preparation of Biotinylated CD1a, b and c

Human CD1a, b, and c were produced as single‐chain constructs with β‐2 microglobulin N‐terminally fused to the heavy chain via a flexible glycine‐serine linker. Expression cassettes were cloned in‐frame with C‐terminal Avi‐tag and His6 tag in pCDNA3.1 encoding an additional BirA Ligase. Protein expression was performed using the Expi293 Expression System (ThermoFisher Scientific). Soluble CD1a, b, and c proteins were purified using nickel affinity and size exclusion chromatography.

#### 3.2.5 Preparation of Biotinylated CD1d‐αGalCer Monomers

KRN7000 (α‐galactosyl‐ceramide/αGalCer)‐CD1d/β2‐microglobulin complexes were generated by in vitro refolding, as previously described [21], biotinylated via an engineered BirA motif on the C‐terminus of CD1d, and further purified by size exclusion chromatography.

#### 3.2.6 Equilibrium Binding by SPR

Binding analysis was performed using a BIAcore T200 equipped with a CM5 sensor chip (Cytiva) at 25°C in PBS containing 0.005% Tween 20 (Merck). Streptavidin was covalently coupled to the chip surface via amine‐reactivity and biotinylated ligands (pMHCI, MR1, CD1) captured, after which unoccupied sites were blocked with soluble d‐biotin. Subsequently, a serial dilution of each CD8‐Fc fusion or mTCR was sequentially injected over the chip. Steady‐state responses were determined from reference‐subtracted data using BIA evaluation (Cytiva) and plotted as a function of analyte concentration using GraphPad Prism (GraphPad Software). Equilibrium dissociation constants (K_D_) were calculated using GraphPad Prism (GraphPad Software) to perform nonlinear curve fit (*Y* = *B*_max_*X/(K_D_ + *X*) to the data assuming 1:1 Langmuir binding (AB = B*AB_MAX_/(K_D_ + *B*)). For a selected subset of interactions, measurements were performed using CD8‐Fc fusions as immobilized ligands. In this instance, Protein A (Sigma‐Aldrich) was covalently coupled to the chip surface via amine‐reactivity and Fc‐fusions captured to ∼1000 RU. Subsequently, a serial dilution of each pMHCI was injected over the chip. Data analyses were performed as described above.

#### 3.2.7 Structural Modeling of CD8 Interactions

Structural modeling was performed using PyMOL [22] by superposing the heavy chains of the MHCI molecule from the complex structure of CD8‐HLA‐A2 [15] with available structures of the other MHCI [23, 24, 25] and unconventional MHC molecules included in this study [26, 27, 28, 29, 30].

### 3.3 Generation of Peptide Specific T‐cells

Peptide‐specific CD8^+^ T‐cell clones were generated from PBMCs from healthy individuals with at least one copy of the relevant HLA class I allele, as previously described [31]. Briefly, T‐cells were stimulated by autologous APCs exogenously loaded with peptide and expanded in culture for up to 3 weeks. T‐cell cultures were then screened for specificity in an IFNγ ELISPOT assay before FACS sorting for T‐cell clones based on either CD137 and CD25 expression following re‐activation by the relevant peptide or binding of the relevant fluorescently labeled pMHCI multimer. TCR sequences were identified for specific T‐cell clones from extracted mRNA.

### 3.4 Preparation of Soluble mTCRs

Production of soluble mTCRs has been previously described [32]. TCR α‐ and β‐chain encoding DNA was PCR amplified from cDNA prepared from peptide‐specific T‐cell lines and cloned into bacterial expression vectors. TCR chains were expressed in *E. coli* and were refolded from denatured inclusion bodies as soluble disulfide‐linked heterodimeric mTCRs and purified by anion exchange and size exclusion chromatography.

## Author Contributions

Ben de Wet performed experiments and wrote the manuscript. Robert Alan Simmons prepared reagents and critically reviewed the manuscript. Richard Suckling prepared reagents and critically reviewed the manuscript. Rita Szoke‐Kovacs prepared reagents and critically reviewed the manuscript. Salah Mansour prepared reagents and critically reviewed the manuscript. Marco Lepore conceived an experimental design and critically reviewed the manuscript. David K. Cole conceived experimental design, performed structural modeling, and critically reviewed the manuscript. Jakub Jaworski performed experiments and critically reviewed the manuscript. Alexandra Chapman prepared reagents and critically reviewed the manuscript. Milos Aleksic conceived an experimental design and critically reviewed the manuscript.

## Conflicts of Interest

The authors declare no conflicts of interest.

### Peer Review

The peer review history for this article is available at https://publons.com/publon/10.1002/eji.202451230.

## Supporting information



Supporting Information

## Data Availability

The data that support the findings of this study are available from the corresponding author upon reasonable request.
