# Supplementary material for: Characterization of Human CD8αβ Interaction With Classical and Unconventional MHC Molecules
Source: Eur J Immunol. 2024 Dec 20;55(1):e202451230. doi: 10.1002/eji.202451230 (PMC11739670; doi:10.1002/eji.202451230)
Supplement: Supplementary file 1 — Supporting Information [file EJI-55-e202451230-s001.pdf]

Characterization of human CD8αβ interaction with classical and unconventional MHC molecules

Ben de Wet, Robert Alan Simmons, Richard Suckling, Rita Szoke-Kovacs, Salah Mansour, Marco Lepore, David K. Cole, Jakub Jaworski, Alexandra Chapman, Milos Aleksic.

SUPPLEMENTARY INFORMATION

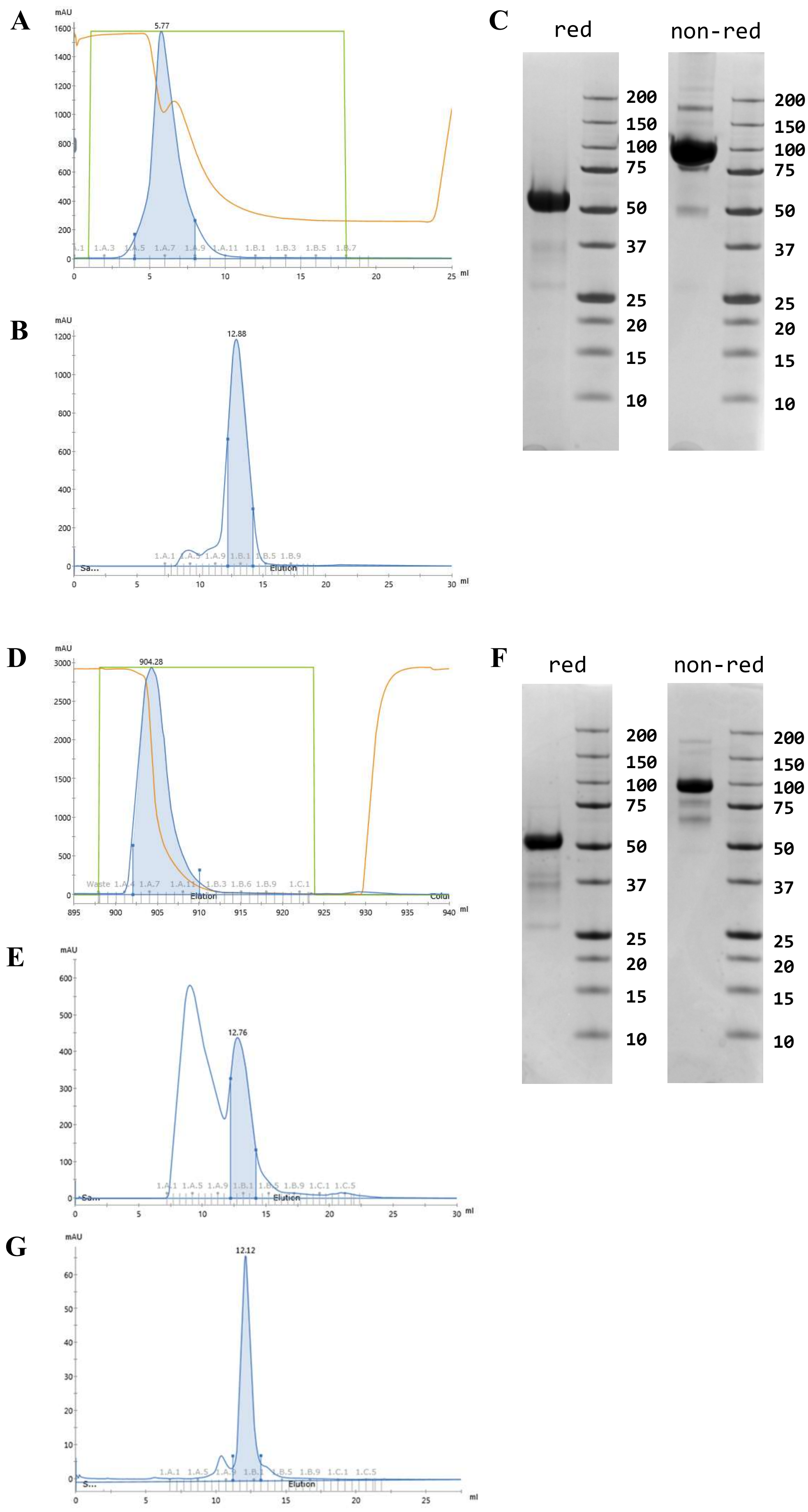

Figure S1: Purification of mammalian expressed CD8αα-Fc and CD8αβ-Fc.
CD8αα-Fc (A,B&C) and CD8αβ-Fc (D,E&F) were purified using Protein A affinity chromatography (A&D), followed by gel filtration (B&E). Purity was further assessed by SDS-PAGE under reducing and non-reducing conditions (C&F). A sample of purified protein was incubated at 4°C for 7d before subsequent gel filtration to confirm absence of cumulative aggregation (G).

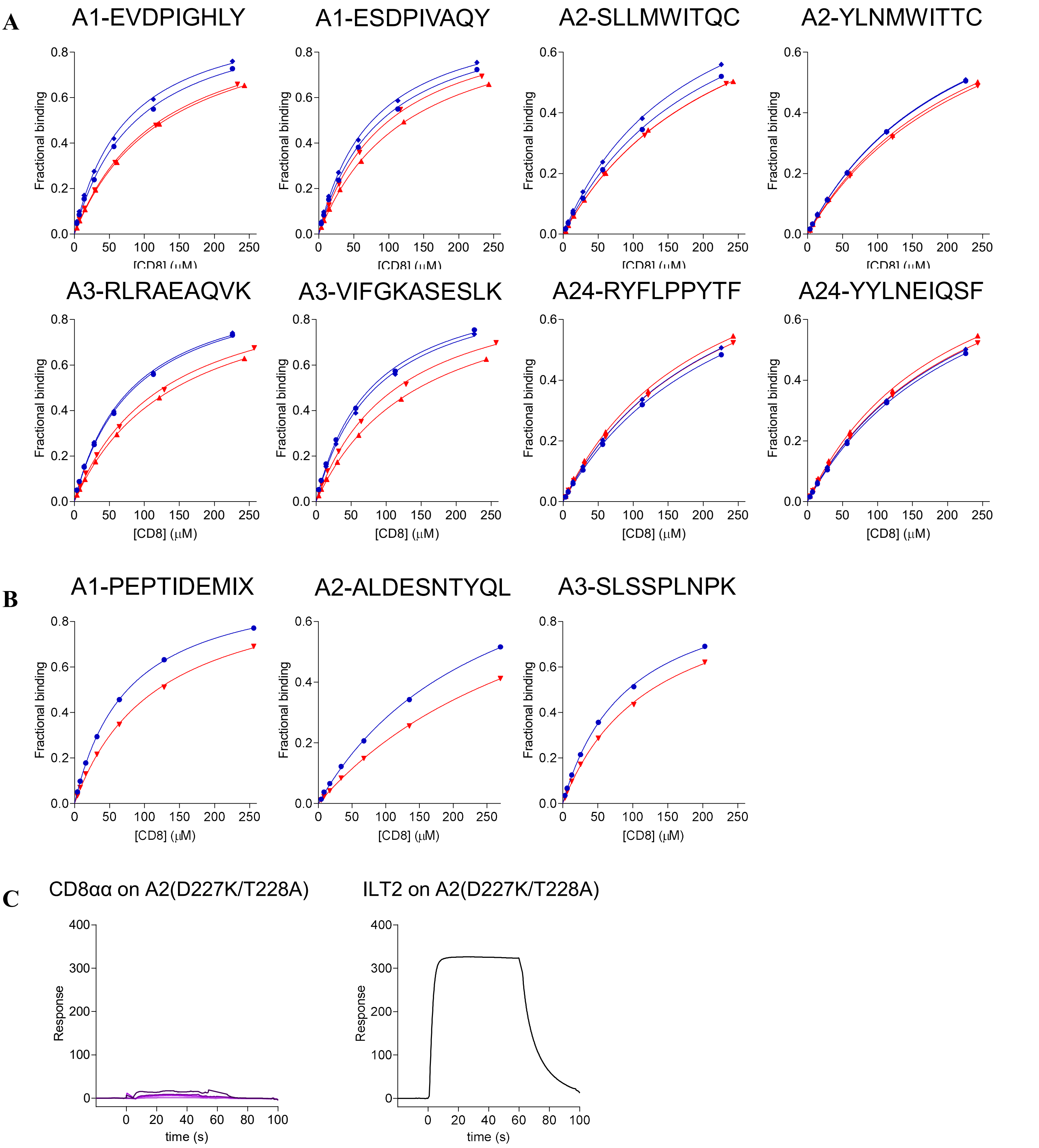

Figure S2: Binding of CD8 to pMHCI alleles.
(A) Equilibrium binding of CD8αα-Fc (blue) and CD8αβ-Fc (red) as function of analyte concentration to several MHCI alleles and peptides measured by SPR at 25ºC. Equilibrium dissociation constants (K_D) were calculated using GraphPad Prism (GraphPad Software) to perform nonlinear curve fit ($Y = B_{max}*X/(K_D + X)$ to the data assuming 1:1 Langmuir binding ($AB = B*AB_{MAX}/(K_D + B)$). Affinity measurements reported in Table S1. Duplicate experimental repeats shown. (B) Reversal of analyte and ligand produces similar binding by SPR. Equilibrium binding of a number of MHCI alleles to CD8αα-Fc (blue) and CD8αβ-Fc (red) as function of analyte concentration. A1-PEPTIDEMIX consists of HLA-A1 refolded with a mixture of the following common peptide (YSDKYGLGY, DTDHYFLRY, STDHIPILY, HSDPSILGY, KSDVHLNFY, HTDILKEKY, DLDSRVLYY, IAD(M/M(O))GHLKY, LTELPDWSY, ASDPFFRHY, ETEKDFSRY, GTVYEDLRY, YLDDPDLKY, TSDELQFGY, SSEQTFMYY, SSDNIMVQY, SSDANPVRY, VSDKTLSFY, ATDDVAQIY, NTDPHSQEY). Data from a single experiment shown. (C) Response curves showing no binding of CD8αα-Fc to CD8-null variant of A2 (D227K/T228A) (purple) despite ILT2 (1 μM) binding to CD8-null variant (black). Data from a single experiment shown.

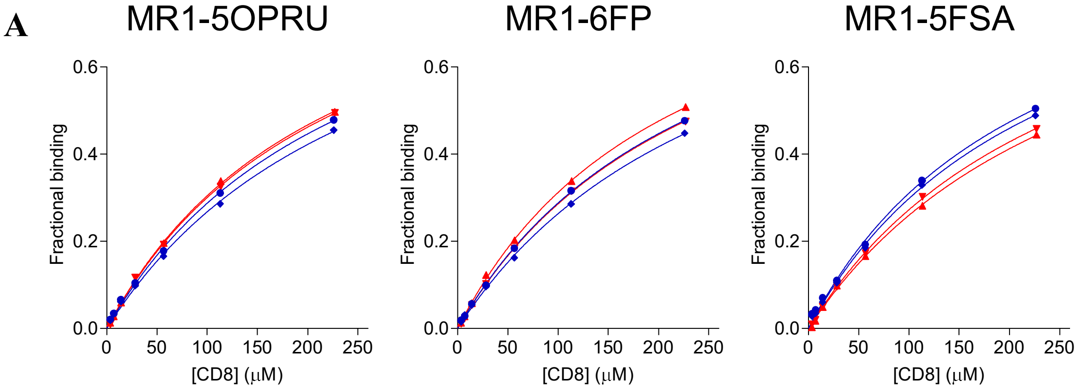

Figure S3: Binding of CD8 to MR1.
(A) Equilibrium binding of CD8αα-Fc (blue) and CD8αβ-Fc (red) as function of analyte concentration to MR1 loaded with various ligands measured by SPR at 25ºC. Duplicate experimental repeats shown.

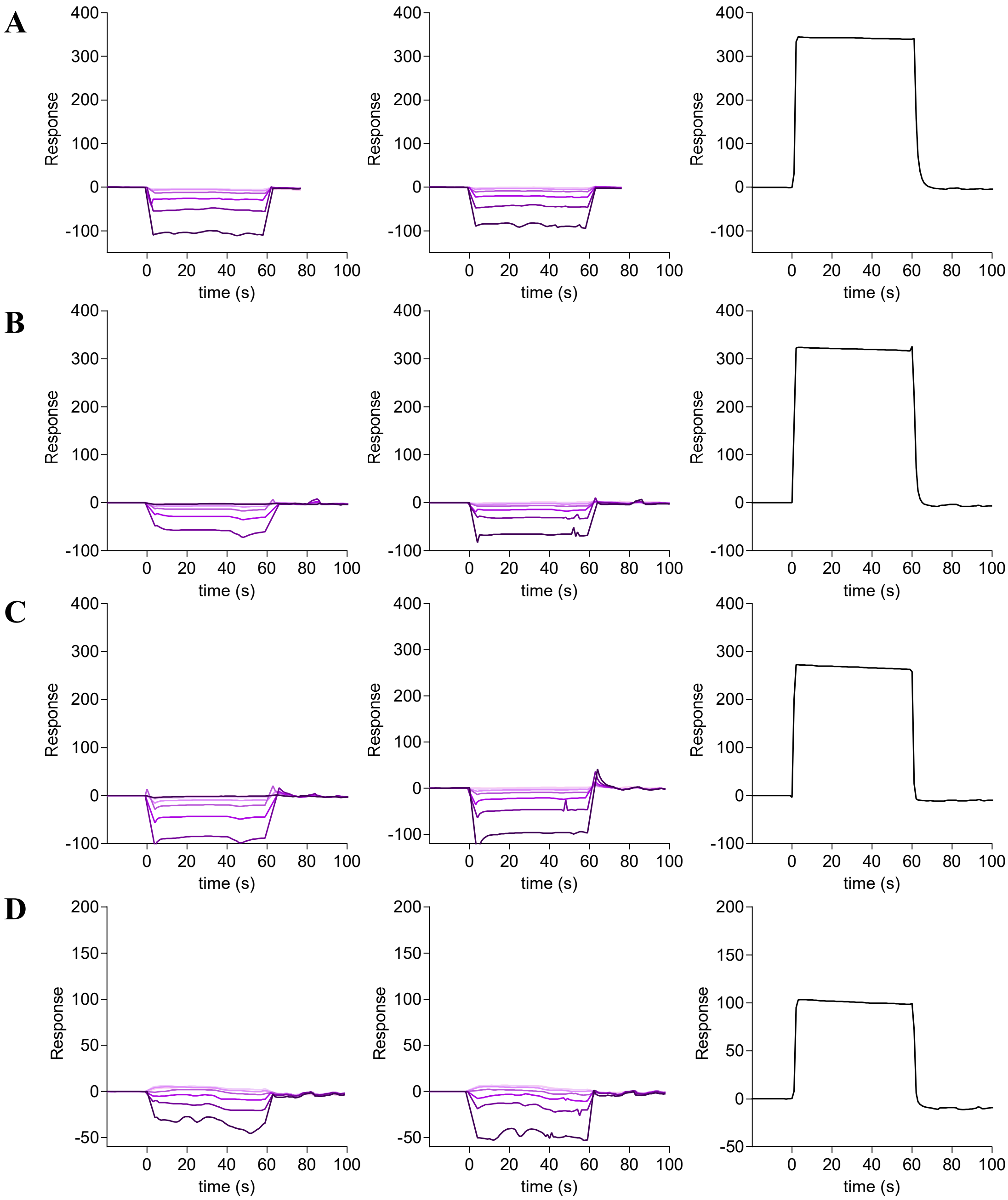

Figure S4: Lack of binding of CD8 to CD1.
SPR response curves showing no binding of CD8αβ-Fc (left-hand column, maximum injection 270 μM) or CD8αα-Fc (middle column, maximum injection 203 μM) to CD1a (A), CD1b (B), CD1c (C) or CD1d (D) despite ILT2 binding (40 μM) to all CD1's (right-hand column). Data from a single experiment shown.

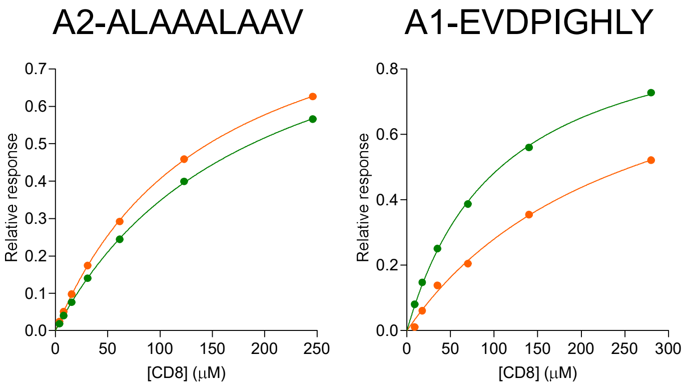

Figure S5: Binding of CD8 to HLA-A2 and –A1 mutants.
(A) Equilibrium binding of CD8αβ-Fc to A2-ALAAALAAV and A1-EVDPIGHLY wild-type (green) and complementary α2-domain triple mutants (A2-H114R/Y116D/K127 and A1-R114H/D116Y/N127K, orange) measured by SPR at 25ºC as function of analyte concentration. Equilibrium dissociation constant for binding to A2-ALA was 189 μM compared to 146 μM for binding to A2(H114R/Y116D/K127)-ALA, while conversely the equilibrium dissociation constant for binding to A1-EVD was 107 μM compared to 257 μM for binding to A1(R114H/D116Y/N127K)-EVD. Data from a single experiment shown.

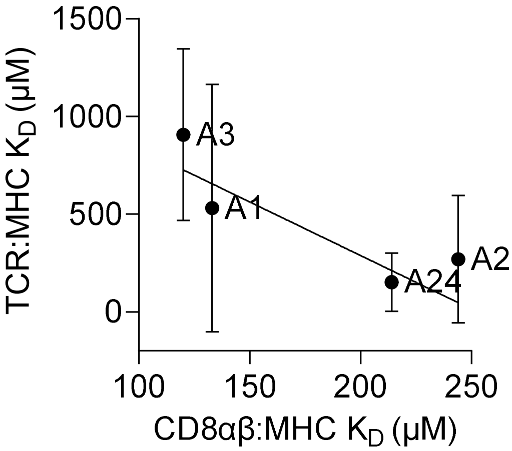

Figure S6: TCR:pMHC affinity as a function of TCR:CD8αβ affinity. TCR:pMHC affinity plotted as a function of TCR:CD8αβ affinity. A variance-weighted linear regression line fitted to pMHC:TCR KD as a function of CD8αβ:TCR KD had slope of -5.5 with coefficient of determination (R2) of 0.71. TCR:pMHC affinity shown as average ± S.D. of multiple single measurements and TCR:CD8αβ affinity shown as average of four experimental repeats.

	Analyte			
	CD8αβ-Fc		CD8αα-Fc	
Ligand	K_D (μM)		K_D (μM)	
A1-EVDPIGHLY	124	129	88	75
A1-ESDPIVAQY	101	126	89	77
A2-SLLMWITQC	238	238	210	180
A2-YLNMWITTC	255	244	221	219
A3-RLRAEAQVK	127	144	86	83
A3-VIFGKASESLK	115	147	85	78
A24-RYFLPPYTF	222	205	243	221
A24-YYLNEIQSF	223	205	237	226
MR1-5OPRU	228	233	248	274
MR1-6FP	250	220	248	280
MR1-5FSA	267	285	222	236

Table S1: CD8 binding to different MHCI alleles and peptides and MR1 loaded with different cargo. Equilibrium dissociation constants (K_D) of CD8αβ-Fc and CD8αα-Fc binding to a number of MHCI alleles with different peptides and MR1 loaded with different cargo, measured by SPR. Measurements were performed at 25ºC and K_D-values in μM of duplicate experimental repeats are shown.

	Ligand	
	CD8αβ-Fc	CD8αα-Fc
Analyte	K_D (μM)	K_D (μM)
A1-PEPTIDEMIX	118	76
A2-ALDESNTYQL	387	255
A3-SLSSPLNPK	126	93

Table S2: HLA alleles binding to CD8αβ-Fc orCD8αα-Fc. Equilibrium dissociation constants (K_D) of a number of MHCI alleles binding to CD8αβ-Fc and CD8αα-Fc measured by SPR. Measurements were performed at 25ºC and K_D-values in μM. A1-PEPTIDEMIX consists of HLA-A1 refolded with a mixture of the following common peptide (YSDKYGLGY, DTDHYFLRY, STDHIPILY, HSDPSILGY, KSDVHLNFY, HTDILKEKY, DLDSRVLYY, IAD(M/M(O))GHLKY, LTELPDWSY, ASDPFFRHY, ETEKDFSRY, GTVYEDLRY, YLDDPDLKY, TSDELQFGY, SSEQTFMYY, SSDNIMVQY, SSDANPVRY, VSDKTLSFY, ATDDVAQIY, NTDPHSQEY). Data from a single experiment shown.
